# Identifying risk factors for 30-day readmission events among American Indian patients with diabetes in the Four Corners region of the southwest from 2009 to 2016

**DOI:** 10.1371/journal.pone.0195476

**Published:** 2018-08-02

**Authors:** Caroline King, Sidney Atwood, Mia Lozada, Adrianne Katrina Nelson, Chris Brown, Samantha Sabo, Cameron Curley, Olivia Muskett, Endel John Orav, Sonya Shin

**Affiliations:** 1 Mel and Enid Zukerman College of Public Health, University of Arizona, Tucson, Arizona, United States of America; 2 Community Outreach and Patient Empowerment (COPE), Gallup, New Mexico, United States of America; 3 Division of Global Health Equity, Brigham and Women’s Hospital, Boston, Massachusetts, United States of America; 4 Gallup Indian Medical Center, Gallup, New Mexico, United States of America; 5 Health Promotion Sciences Department, University of Arizona, Tucson, Arizona, United States of America; 6 Harvard School of Public Health, Boston, Massachusetts, United States of America; 7 Dept. of Global Health & Social Medicine, Harvard Medical School, Boston, Massachusetts, United States of America; VU university medical center, NETHERLANDS

## Abstract

**Objective:**

The objective of this study was to identify risk factors for 30-day readmission events for American Indian patients with diabetes in the southwest.

**Research design and methods:**

Data from patients with diabetes admitted to Gallup Indian Medical Center between 2009 and 2016 were analyzed using logistic regression analyses.

**Results:**

Of 2,660 patients, 394 (14.8%) patients had at least one readmission within 30 days of discharge. Older age (OR (95% CI) = 1.26, (1.17, 1.36)), longer length of stay (OR (95% CI) = 1.01, (1.0001, 1.0342)), and a history of substance use disorder (OR (95% CI) = 1.80, (1.25, 2.60)) were risk factors for 30-day readmission. An American Indian language preference was protective against readmission.

**Conclusions:**

Readmission events are complex and may reflect broad and interwoven disparities in community systems. Future research should work to support community-defined interventions to address both in hospital and external factors that impact risk factors for readmission.

## Background

### Introduction

Hospital readmissions can reflect challenges in healthcare system quality, but understanding the root causes of hospital readmissions can be complex [1]. In marginalized communities, hospital systems exist within the framework of the disparities that exist for patients. Causes of readmission events may not be limited to inpatient care delivery or discharge care, but are likely also impacted by social and economic systems that exist before and after a patient’s hospital stay [2].

The Indian Health Service (IHS) provides healthcare to 2.2 million American Indians and Alaska Natives in the United States [3]. In 2016, the Centers for Medicare and Medicaid Services incorporated IHS into its national Hospital Improvement and Innovation Network to support IHS’s goal to reduce hospital readmissions by 12% through 2019 [4]. CMS’s work primarily looks to improve the quality of care delivered through providing technical resources, opportunities for collaboration and training to healthcare systems to address readmissions [4].

In 2015, twelve IHS facilities located in five states had more than 1,000 hospital discharges [5]. Two of these states, New Mexico and Arizona, are located in the United States southwest and are spanned by the Navajo Nation (which also covers portions of southern Utah). The largest American Indian tribe by population in the United States, the Navajo Nation has over 300,000 tribal members, approximately 170,000 of whom live on the reservation [6, 7]. Diabetes Mellitus (DM) is prevalent on the reservation, with 25,000 tribal members living with diabetes and an additional 75,000 who are prediabetic [8].

Past literature has identified increased readmission rates for American Indian patients [9] and separately, for patients with diabetes [10–13]. The objective of this study was to identify risk factors for 30-day readmission events for American Indian patients with diabetes in the southwest.

## Research design and methods

### Study site

This analysis was completed by the research team at the Community Outreach and Patient Empowerment (COPE) Program in Gallup, New Mexico. COPE is a 501c3 non-profit organization located in Gallup, New Mexico, as well as a sister organization of Partners in Health, a global healthcare organization that works primarily with community health workers globally to strengthen healthcare systems [14]. COPE has worked in partnership with the Navajo Nation since 2009. The data for this project was abstracted for analysis in six of the eight major health facilities comprising the Navajo Area Indian Health Service (IHS) network.

COPE has two community advisory panels that guide the organization’s mission and research plans. The Community Health Advisory Panel (CHAP) is comprised of patients with diabetes, their family members, and Community Health Representatives. The COPE Advisory Group (CAG) is comprised of Community Health Representative supervisors, physicians, nurses, hospital educators, IT personnel, and other healthcare workers who contribute to healthcare systems on the Navajo Nation. Both CHAP and CAG provide input on all research including study design, data interpretation, and generation of additional research ideas and recommendations based on research findings. This project was approved by the Navajo Nation Human Research Review Board and Partners Healthcare Institutional Review Board.

### Study design and setting

This retrospective cohort study includes patients with diabetes mellitus admitted for any reason to the Indian Health Service (IHS) facility Gallup Indian Medical Center (GIMC) in Gallup, New Mexico, between January 1^st^, 2009 and May 31^st^, 2016. GIMC is a health facility run by the IHS that primarily serves American Indian patients. GIMC has 99 in-patient beds and facilitates 250,000 outpatient and 5,800 inpatient visits per year [15].

### Data source

The RPMS system is an Electronic Health Record system that allows for the abstraction of clinical data at IHS facilities [16]. Data for this project was abstracted from the Resource and Patient Management System (RPMS) system at Gallup Indian Medical Center, Northern Navajo Medical Center, Tsehootsooi Medical Center, Chinle Comprehensive Health Care Facility, Crownpoint Health Care Facility, and Kayenta Health Center during the summer of 2016. All data were de-identified and the resulting database was used for study analysis.

### Study participants

Participants had a diabetes mellitus diagnosis via ICD 10 coding between 2009 and 2016 and were admitted at least once to GIMC during the same period. The first admission event after a diabetes diagnosis was used to evaluate covariates relevant to the study question. There were no age restrictions on cohort participation.

### Variables

Covariates were originally selected based on literature review and input from co-authors. A list of covariates considered for inclusion was presented to COPE’s CHAP and CAG members at meetings in January 2017. Covariate lists were modified based on CHAP and CAG suggestions. Both panels identified additional covariates that may contribute to readmission likelihood after a discharge, including barriers to medication access or adherence and family support, that are not well-captured by clinical data at GIMC.

For the purposes of this paper, the readmission event occurs the first time a patient is readmitted within 30 days following a hospital discharge to GIMC or another facility on the Navajo Nation. The index admission event is the admission immediately preceding the readmission. The first admission event is the first admission of the cohort period, regardless of if it is followed by a readmission event.

Binary and categorical covariates evaluated from the time of first admission include sex, language preference (English or Navajo/Zuni), history of substance use disorder, history of chronic kidney disease, history of depression and history of amputation. Continuous covariates evaluated at the time of first admission include age, miles from hometown to GIMC, and number of hospitals in Navajo previously visited.

Continuous covariates evaluated from the index admission include length of stay (days). Finally, binary covariates evaluated from the index admission include if the patient was admitted on a weekend or discharged on a weekend.

The primary outcome was readmission for any reason within 30 days of a discharge during the study period.

### Model building specifications

To avoid overfitting, we used a ten-to-one ratio of mortality events to degrees of freedom [17]. All models were fit to fall within the parameters of this ratio.

### Quantitative variables

Patients with a documented history included in a patient’s problem list of alcohol use disorder or, separately, illicit substance use disorder per ICD 9 and 10 codes at the time of first admission were combined into a single covariate indicating substance use disorder. Age was categorized into ten-year age groups (1–10, 11–20, etc.). Patients speaking Zuni were combined with patients speaking Navajo to account for the small sample size of Zuni speaking patients. Patients without a language preference indicated were assumed to be English speaking.

### Statistical methods

#### Primary analysis

Logistic regression was used to model potential associations between covariates and readmission risk.

#### Missing data

Of the covariates included in testing, we anticipated little potential missingness as only nearly complete variables were originally considered for study inclusion. Complete case analysis was used for covariates missing less than 10% of data with the exception of language. CAG members shared that missing language most likely indicated that patient was English speaking. As such, patients without a language preference indicated were assumed to be English speaking.

#### Primary analysis assumptions and model testing

Observations for this study are independent. Covariates were examined for multi-collinearity using a correlation coefficient of 0.80; covariates with a correlation coefficient of greater than 0.80 were considered to be collinear. A Hosmer-Lemeshow test was carried out for the final regression models, noting that a p value of <0.05 indicates the model does not fit the data well. Linearity in the log-odds was determined using Lowess scatter plots of the binary readmission variable at 30 days with each continuous variable (age, miles, number of hospitals visited, and length of stay).

#### Sensitivity analysis

Sensitivity analyses for the 30-day readmissions models were conducted. Influential observations were identified using Pregibon’s Delta-Beta statistic at values greater than 0.2 and removed.

## Results

### Study participants

**Fig 1:**
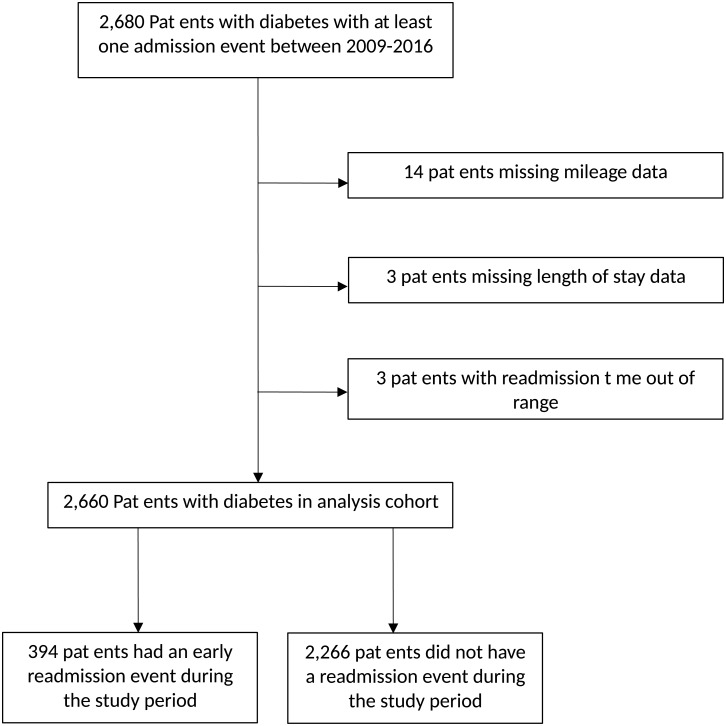
Participant flowchart for cohort participation, 2009–2016 at Gallup Indian Medical Center.

Between 2009 and 2016, 2,680 patients with diabetes were admitted to GIMC (Fig 1). 2,660 had complete data (after dropping patients missing length of stay, mileage, and patients with data errors, Table 1). Of these, total of 394 (14.8%) patients had at least one readmission within 30 days of discharge.

**Table 1:** Baseline characteristics and missing data for American Indian patients with diabetes admitted to GIMC between 2009 and 2016.

Characteristic	Readmitted ≤ 30 days (n = 394)	Not readmitted ≤30 days (n = 2,266)
**Age (years)**	61.5 (17.2)	57.2 (16.4)
<10 years old	1 (0.3)	0
10 to 19 years	2 (0.5)	13 (0.6)
20 to 29 years	9 (2.3)	112 (4.9)
30 to 39 years	35 (8.9)	242 (10.7)
40 to 49 years	69 (17.5)	393 (17.3)
50 to 59 years	64 (16.2)	493 (21.8)
60 to 69 years	63 (16.0)	471 (20.8)
70 to 79 years	90 (22.8)	357 (15.8)
80 to 89 years	53 (13.5)	160 (7.1)
90 to 99 years	8 (2.0)	24 (1.1)
>100 years old	0	1 (0.04)
**Sex** Male	194 (49.2)	1040 (45.9)
**Language Preference** Navajo/Zuni	104 (26.4)	624 (27.5)
**Length of Stay (days)**	5.5 (5.5)	4.7 (5.7)
**Number of sites visited**	1.24 (0.51)	1.23 (0.48)
**Miles from GIMC**	30.9 (56.8)	37.2 (60.5)
**Substance Use**	47 (11.9)	196 (8.6)
**Chronic Kidney Disease**	46 (11.7)	179 (7.9)
**Depression**	10 (2.5)	37 (1.6)
**Amputation**	9 (2.3)	27 (1.2)
**Weekend admit**	87 (22.1)	541 (23.9)
**Weekend discharge**	93 (23.6)	480 (21.2)

Values shown are n(%) or mean (standard deviation)

### Main results

For 30 day readmissions, a history of substance use disorder (OR (95% CI) = 1.80, (1.25, 2.60)) older age (OR (95% CI) = 1.26, (1.17, 1.36)), and longer length of stay (OR (95% CI) = 1.01, (1.0001, 1.0342)) were risk factors for readmission (Table 2). Indicating a language preference of either Navajo or Zuni was protective against 30 day readmissions (OR (95% CI) = 0.72, (0.55, 0.93)).

**Table 2:** Odds ratios, 95% confidence intervals, and p-values for covariates included in adjusted logistic regression models to identify risk factors for 30-day readmission models among American Indian patients with diabetes in the Southwest.

	30 day model
	OR (95% CI)
**Age (years)**	1.26 (1.17, 1.36)
**Sex** Male	1.14 (0.91, 1.43)
**Language Preference** Navajo/Zuni	0.72 (0.55, 0.93)
**Length of Stay (days)**	1.02 (1.0001, 1.0342)
**Number of sites visited**	0.99 (0.79, 1.25)
**Miles from GIMC**	0.998 (0.996, 1.0001)
**Substance Use**	1.80 (1.25, 2.60)
**Chronic Kidney Disease**	1.39 (0.98, 1.97)
**Depression**	1.60 (0.78, 3.28)
**Amputation**	1.71 (0.79, 3.74)
**Weekend admit**	0.91 (0.70, 1.18)
**Weekend discharge**	1.21 (0.93, 1.56)

### Sensitivity analysis

Influential points were identified using Pregibon’s delta-beta statistic. Observations with values greater than 0.20 were removed during sensitivity analysis. In the 30-day model, once influential points were removed, length of stay was no longer significant. All other significant covariates have odds ratios within 10% difference of the primary analysis. Higher mileage from hometown to GIMC was protective against readmission (OR (95% CI) = 0.995, (0.992, 0.998)).

## Discussion

The objective of this study was to identify risk factors for 30-day readmission events for American Indian patients with diabetes in the southwest United States. We identified a history of substance use disorder, older age, and a longer length of stay as risk factors for 30-day readmission, while speaking either Navajo or Zuni was protective against readmission. Sensitivity analysis results added living closer to GIMC as a risk factor for 30 day readmissions and removed length of stay.

In the Navajo Nation, disease prevention, incidence and management is shaped by economic and social systems that lie downstream from the historic and engrained effects of colonization [18–20]. Alongside diabetes, this is likewise true for the aforementioned comorbidities included in this study [18, 20]. While readmissions are commonly attributed to healthcare system quality, healthcare system functioning itself in Navajo exists within the context of disparities. While the IHS, created in 1955, forced reliance on and belief in the delivery on Western medicine at the expense of traditional knowledge, the IHS today remains underfunded and understaffed [21]. In 2015, the United States Congress allocated $3,688 in health expenditures within IHS per federally recognized tribal member, while other federally funded healthcare programs received an allocation of $9,523 per person [3]. In 2016, IHS’s vacancy rate for physicians and nurse practitioners remained at 25% [22]. Thus, considering readmissions within this framework is complex. Past research has shown benefit to targeted inpatient hospital interventions to improve discharge planning and reduce readmissions [23]. Within the framework of marginalized communities, however, it is likely that additional, systems-level work is required to eliminate preventable readmissions.

A clear example of the complex and overlapping areas of marginalization and readmissions is the identification of substance use disorders as a risk factor for 30-day readmissions in Navajo. Substance use disorders are prevalent in Navajo [24] for many reasons, including historical inequities that have made building back of local culture, economic and social systems until recently, slow [20]. Comprehensive inpatient treatment options for substance use disorders near the reservation include six tribal or IHS behavioral health or alcohol treatment centers, however, difficult roads, distance, and other barriers can make them not easily accessible to people living on the reservation [25]. In order to continue to address the complex dynamics of readmission for patients with both diabetes and a substance use disorder community-defined solutions to treatment options, prevention, and patient and family support must be used.

As compared to non-marginalized communities, American Indian patients with diabetes may benefit most from inpatient efforts to reduce readmissions that take into context the barriers patients experience after discharge, as well as an understanding from practitioners of the limitations of systems that may have contributed to a patient’s initial presentation. Gallup Indian Medical Center has, in recent years, worked with the COPE Program to use culturally-specific training materials created for patients with diabetes to improve patient education at the time of discharge. Additional support for targeted programs such as this could contribute to reducing hospital readmissions, or at the very least, empowering patients to better understand and potentially address aspects of their illness within their control. Future qualitative work should also seek to understand patient experiences after hospital discharge, particularly among patients with one or more comorbidities, to continue to identify specific ways hospital care can be tailored to meet patient needs.

Separately, this paper also suggests that patients who have an indicated language preference of Navajo or Zuni may be protected against hospital readmissions. Research with Navajo Nation Community Health Representatives has shown that trust in healthcare delivery is improved through recognition of a patient’s tradition and culture [26]. Two possible explanations for the protective effect of language are that first, in documenting a patient’s preferred language other than English, patients experience improved trust with the healthcare system which may improve healthcare outcomes, or second, that the ties to tradition and culture are themselves protective against readmission because of a stronger sense of personal health.

Results from this study cleanly map onto past published research related to readmissions among patients with diabetes. Across hospital readmission studies of patients with diabetes, age and length of stay have both been associated with increased risk of readmission, as has living closer to a hospital [10–12, 27–30]. Increased comorbidities burden has also broadly been associated with increased risk of readmission [10, 12, 30]. Similarly, in studies with patients who primarily do not speak English, access to interpretive services has been associated with reduced readmission risk [31]. This may be relevant if patients who indicated a preferred language of Navajo or Zuni had improved access to translator services; future qualitative research could shed light on how hospital systems manage care for patients who do not primarily speak English.

The paper was strengthened by the use of a dataset comprised nearly entirely of data from American Indian patients to help understand readmission risk factors for this population. Additionally, stakeholders from COPE’s CHAP and CAG boards helped shape this paper’s methodology and analysis.

This paper was limited by the choice to use covariates either at the time of the start of the cohort period or present any time before the admission prior to the readmission occurred. Future research should consider including additional covariates from the preceding admission event including hemoglobin A1C values, history of primary care empanelment, and others. Additionally, future research would benefit from considering insulin therapy as a covariate at the time of first admission, as well as additional disease characteristics including neuropathy, retinopathy, and others.

Readmission events are complex and may reflect broad and interwoven disparities in community systems, which impact patient health. Future research should work to support community-defined interventions to address both in hospital and external factors that impact risk factors for readmission.
